# Comparative Evaluation of Midventral and Flank Laparotomy Approaches in Goat

**DOI:** 10.1155/2014/920191

**Published:** 2014-08-24

**Authors:** A. A. Abubakar, R. A. Andeshi, A. S. Yakubu, F. M. Lawal, U. Adamu

**Affiliations:** ^1^Department of Veterinary Surgery and Radiology, Usmanu Danfodiyo University, Sokoto 2346, Nigeria; ^2^Department of Theriogenology and Animal Production, Usmanu Danfodiyo University, Sokoto 2346, Nigeria

## Abstract

The aim of the study was to compare two laparotomy approaches (flank and midventral). Ten (*n* = 10) apparently healthy goats of different breeds and sex, average age of 12 ± 2.1 months, and average weight of 13.4 ± 2 kg were used for the investigation. The goats were randomly divided into flank and midventral groups, each group comprising five goats (*n* = 5). Standard aseptic laparotomy was performed under lumbosacral epidural anaesthesia with mild sedation. Postsurgical wound score showed significant difference (*P* < 0.05) in erythema at 18–24 hours and 10–14 days after surgery between the two approaches; significant difference of dehiscence between the two groups was also recorded at 10–14 days after surgery. Total white blood cells (WBC) and lymphocytes counts were significantly different (*P* < 0.05) at the first and second week after surgery. There was significant difference of platelets critical value and platelets dimension width at the first and second week after surgery. Significant difference of packed cells volume between the two approaches was also recorded one week after surgery. It was concluded that midventral laparotomy approach can be conveniently and safely performed under aseptic precautions without fear of intra- and postoperative clinical problems.

## 1. Introduction

Laparotomy in goat is an invasive surgical procedure into the abdominal cavity that allows visual examination of abdominal organs and documentation and correction of certain pathologic abnormalities observed [1, 2]. Generally, it constitutes the single largest group of surgical operations carried out in ruminants [3, 4]. Laparotomy is indicated for exploration of abdominal and pelvic cavities and other surgical procedures involving abdominal and pelvic organs; other specific indications are caesarean section, embryo transfer to produce transgenic goats, ovariectomy, rumenotomy, abomasotomy, ventral abdominal herniorrhaphy, intestinal resection, anastomosis, and cystotomy [5–11]. Two approaches (flank and midventral) have been recognized and are currently in use in both small and large animals surgery; however in ruminants flank approach is the most widely and frequently practiced [1, 2]; due to the fact that surgical site can be visualized and observed from a distance and access healing, it was also reported to have reduced potential risk for evisceration if wound dehiscence is to occur, and the overlapping arrangement of the oblique muscles in the flank helps maintain the integrity of the abdominal wall if wound complication occurs [7].

The flank laparotomy approach is the most widely used among small ruminants surgeons for accessing abdominal and pelvic organs. However, the approach is associated with some challenges: animals tend to rub the surgical site during healing against available solid objects leading to loosening of sutures and subsequently formation of wound dehiscence, prolonged lateral recumbency in goats under anaesthesia is associated with decrease in rumen stasis thereby predisposing the animal to bloat and toxemic lactic acidosis, and the accessibility to the distant organs (far proximal or distal to the point of incision) is also limited [12]. We hypothesized that midventral laparotomy approach could be an alternative to flank laparotomy approach without much intra- and postsurgical complications. To test this hypothesis we compare the surgical wound assessment, intra- and postsurgical assessment, haematological profile, and subjective healing interval of the two laparotomy approaches. The aim of the study was to compare and evaluate flank and midventral laparotomy approaches in goats.

## 2. Material and Methods

Ten (*n* = 10) apparently healthy goats free of any dermatological lesions with average age of 12 ± 2.1 months (mean ± SD), male and female of different breeds, and average weight of 13.4 ± 2 kilograms (mean ± SD) were used for the investigation. The goats were kept at the Usmanu Danfodiyo University Veterinary Teaching Hospital facilities and were conditioned for two weeks during which they were evaluated and stabilized for surgery. During evaluation serial blood sampling was done for comprehensive haematology to ascertain that the goats are fit for surgery and fecal sample was also collected to ascertain the intestinal worms burden. The goats were maintained on daily ration comprising wheat bran, bean husks, ground nut hay, and water* ad libitum*. The goats were randomly grouped into flank (FA) and midventral (MVA) approaches. Five (*n* = 5) goats were allocated to each group.

### 2.1. Surgical Procedure

Feed and water were withdrawn from animals at least 12 hours prior to the surgery. The left flank region of each goat in the FA group was prepared for routine aseptic surgery by clipping the hairs around the proposed surgical site; the site was scrubbed with Purit solution containing chlorhexidine gluconate B. P. 0.3% W/V (Saro Lifecare Limited, Lagos, Nigeria) and rinsed with methylated spirit (Binji Pharmaceutical Company, Sokoto, Nigeria). Regional anesthesia was achieved with plain lignocaine hydrochloride and lignocaine injection B. P. 2% (Sahib Singh Agencies, Mumbai, India) at 4 mg kg^−1^ through lumbosacral epidural anaesthesia as described by [13]. The epidural space was identified by loss of resistance to injection of 1 mL of air after piercing the ligamentum flavum. Mild sedation was achieved using xylazine 20 (xylazine HCl 20 mg mL^−1^, Kepro Holland) at 0.025 mg kg^−1^ intramuscular and atropine sulphate 0.6 mg mL^−1^ (Laborate Pharmaceuticals India) at 0.05 mg kg^−1^ intramuscular as vagolytic agent.

Goats in FA group were placed on right lateral recumbency exposing the left flank. Laparotomy was done according to standard procedure described by [1, 3, 14]. The laparotomy was routinely closed from within outward; muscle layers were closed using Becton chromic catgut of the size of 1/0 and atraumatic 1/2 circle taper point needle (Anhui Kangning Industrial Groups, China) using interrupted horizontal mattress suture pattern with simple interrupted reinforcement. The subcutaneous layer was closed using Becton chromic catgut of the size of 2/0 and atraumatic 1/2 circle taper point needle using simple continuous suture pattern. The skin was closed using Ford interlocking pattern with Agary nylon of the size of 0 and atraumatic 3/8 curved, cutting needle (Agary PharmaceuticalsLtd, Xinghuai, China). In MVA group, the cranial midventral area was prepared for aseptic procedure as described in FA group. Regional anesthesia was also achieved as described in FA group. Each animal was placed on dorsal recumbency exposing the midventral region. Laparotomy was done through linea alba in all female goats with little paramedian incision at the level of prepuce in all the males according to standard procedure described by [1, 3, 4]. The incision was closed routinely in three layers from within outward (linea alba, subcutaneous layer, and skin) with the same suture materials as described in FA group. The linea alba was closed using interrupted horizontal mattress pattern with simple interrupted reinforcement. 5% acetaminophen injection 10 mg kg^−1^ intramuscular (Cadence Pharmaceutical Inc., Ireland) was administered for 3 days after surgery to take care of postoperative pain. Long acting 15% amoxicillin injection 20 mg kg^−1^ (Vetrimoxin) was administered once after surgery.

#### 2.1.1. Surgical Wound Assessment

The clinical appearance of the skin was assessed and scored twice: 18–24 hours and 10–14 days after surgery as described by [15] using 4-point scoring scale, based on the following criteria: discharge, swelling, erythema, and dehiscence.

#### 2.1.2. Haematology

Blood samples were collected from each animal in the two groups through the jugular vein after thorough disinfection of the area with methylated spirit; the sample was collected using 5 mL syringe and needle into EDTA bottles. The samples were collected before surgery as baseline, 18–24 hours after surgery, and subsequently on weekly interval till complete healing when sutures were removed. The samples were analyzed using digital automated haemoanalyser (Full Automated Blood Cell Counter PCE-210, Erma Inc., Tokyo, Japan) according to procedure described [16].

#### 2.1.3. Intra- and Postoperative Complications

Intra- and postsurgical complications were assessed using 3-point scoring system designed by ourselves; parameters considered were intraoperative haemorrhages, postsurgical seroma, incisional hernia, and wound fistula (Table 1).

### 2.2. Subjective Healing Interval

Subjective healing interval was determined by visual observation and taking notes of days of apparent surgical site healing according to [17].

### 2.3. Statistical Analysis

Data generated from the four parameters (surgical wound scoring, haematology, surgical complications, and healing interval) were tabulated and mean and standard deviation were computed in each case. Student's *t*-test was used to compare statistical significant difference between the flank and midventral variables of each parameter at 95% confident interval using GraphPad Instat Statistical software package 2010. *T* value was considered significant when *P* value is less than 0.05.

## 3. Results

### 3.1. Postsurgical Wound Assessment

At 18–24 hours after surgery, there was serous discharge in all groups; the mean discharge scores were (0.80 ± 0.45 and 0.80 ± 0.84) for flank and midventral approaches, respectively. There was no significant difference between the two groups when compared. At 10–14 days after surgery, there was no discharge observed (Table 2).

Midventral group had higher swelling score (2.00 ± 00) in comparison with flank approach (1.8 ± 0.45) and the overall swelling score was higher at 18–24 hours after surgery compared to 10–14 days after surgery (0.50 ± 0.56 and 0.80 ± 0.45) in flank and midventral, respectively (Table 2). There was no significant difference between flank and midventral approach both at 18–24 hrs and at 10–14 days after surgery.

The flank approach at 18–24 hours had higher erythema score (1.40 ± 0.55) when compared with midventral group (0.80 ± 0.45) and there was significant difference (*P* < 0.05) of erythema between the two approaches (Table 2). At 10–14 days after surgery, flank approach had higher erythema score (0.25 ± 0.50) while midventral approach had no erythema record and there was significant difference (*P* < 0.05) between the two approaches.

Dehiscence was not recorded at 18–24 hours after surgery in all the groups; however, at 10–14 days after surgery dehiscence was observed in flank approach with significant difference (*P* < 0.05) between the two groups (Table 2).

### 3.2. Intra- and Postsurgical Complications

Intraoperative haemorrhage score was higher in flank approach (1.4 ± 0.55) when compared with midventral approach (1.00 ± 0.70); there was no significant difference (*P* > 0.05) between the two groups (Table 3). There were no postoperative complications of incisional hernia, seroma, and wound fistula recorded.

### 3.3. Haematological Profiles

There were variations of total white blood cells (WBC) count of the two approaches before surgery, at 18–24 hours, and at the first and second week after surgery; the midventral group had higher WBC value at all the intervals with significant differences (*P* < 0.05) at first and second week after surgery (Table 4). There were slight variations of total granulocytes between the two groups with the midventral group having the higher values at all the intervals, but there is no significant difference between the two groups (Table 4). The lymphocytes values of the two groups also varied and the midventral approache had the highest value. There were significant differences (*P* < 0.05) recorded between the two approaches at first and second week interval: 21.33 ± 8.22 flank approach against 28.32 ± 11.98 midventral approach and 15.20 ± 3.52 flank approach against 25.48 ± 6.00 midventral approach (Table 4). There were also slight variations of monocytes values between the flank and midventral approaches at different timing interval; the midventral had higher values when compared with flank approach but there were no significant differences between the two approaches at any given time interval (Table 4).

The values of the platelets varied slightly between the two approaches, with the midventral approach having a higher value when compared with flank approach, and there was no significant difference between the two approaches at all the timing intervals (Table 5). The platelets critical values varied between the two approaches with the midventral having the higher values; there was significant difference (*P* < 0.05) at second week interval between the flank and midventral approach (0.15 ± 0.04 against 0.25 ± 0.08), respectively (Table 5). The mean platelets volumes also showed slight variations between the two groups, but there was no significant difference between the groups at any of the timing intervals; the midventral approach had higher values when compared with the flank approach (Table 5). The platelets dimension width values were slightly higher in midventral approach compared to flank approach and a significant difference (*P* < 0.05) was recorded between the two approaches at 18–24-hour interval (Table 5).

The packed cells volume of the two approaches showed slight variations with the midventral approach having the higher PCV values when compared with the flank approach. There was significant difference (*P* < 0.05) recorded at one week interval between the two approaches (Table 6). There were no significant differences (*P* > 0.05) between the two approaches in all other erythrocytic indices (red blood cells count, haemoglobin, mean corpuscular volume, mean corpuscular haemoglobin, mean corpuscular haemoglobin concentration, and red blood cells distribution width). However, the values of midventral approach are higher at different timing intervals in all other erythrocytic indices (Table 6).

### 3.4. Subjective Healing Interval

**Figure 1:**
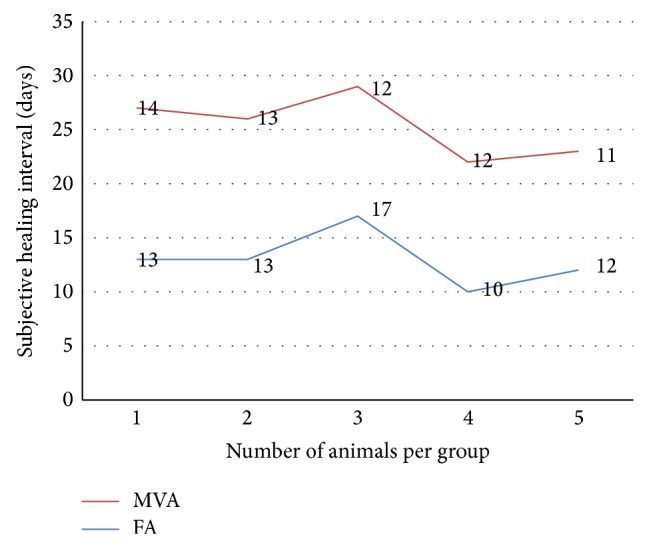
Subjective healing interval (days) of the animals flank (FA) and midventral (MVA) approaches.

The mean subjective healing intervals were 13.0 ± 1.14 and 12.4 ± 0.5 for flank and midventral approach. Midventral approach had lower mean healing intervals in days compared to the flank approach. There was no significant difference (*P* = 0.643) between the two groups when compared (Figure 1).

## 4. Discussions

Laparotomy is commonly indicated either for exploratory purposes when clinical diagnosis is uncertain or for therapeutic surgical intervention when specific diagnosis has been made [2]. Flank approach is the most commonly practiced technique among large animal surgeons with the animal under local anaesthesia [18]. Ventral paramedian or midventral laparotomy approach is an alternative practice by few large animal surgeons that necessitates the animal placement in dorsal recumbency. The two main indications in bovine are ventral abomasopexy and cesarean section, in which it offers advantages in the delivery of oversized or emphysematous fetuses and in complicated deliveries, including uterine tears [12, 19].

Surgical wound assessment showed significant difference of erythema both at 18–24 and at 10–14 days after surgery with flank approach having the highest erythema score and this could be due to surgical trauma elucidated by the traumatic surgical instruments on the soft tissue in the course of surgery; this is because the flank region has three layers of abdominal muscles that have to be passed through before getting access into the abdominal cavity in comparison with midventral approach through linea alba aponeurosis (ligament) which is passed through before gaining access to abdominal cavity; the ligament poorly responds to pressure of traumatic surgical instruments which brought about the less erythematous response. The high erythema score recorded in flank approach could also be a result of abdominal muscles tissue response to absorbable suture materials used for apposing the muscles mass which is more bulky than that of midventral approach. The overall scoring showed higher erythema earlier before surgery at 18–24 hours and this finding is consistent with the studies conducted by [15, 17] where significant differences among the variables were observed.

Dehiscence was also observed in the flank approach at 10–14 days after surgery with significant difference when compared with midventral approach; this could be a result of scratching the surgical site (flank) with available objects in the pen as a result of tissue irritation in the course of healing process. It could also be due to self-mutation with horn of hind limbs in response to tissue irritation. Dehiscence score was by far less in midventral approach due to lesser chances of scratching and self-mutilation around the region. Our finding was contrary to that of [15], which recorded no dehiscence in a similar study using canine species, and that of [17], which recorded mild dehiscence both at 18–24 hours and at 10–14 day after surgery but without significant difference in a similar study using caprine species.

The intraoperative hemorrhage score recorded was higher in the flank approach compared with the midventral approach, though without significant difference; this could be a result of high vascular channels available in the abdominal muscle mass when compared to poor vasculatures associated with tendons and ligament in the linea alba. This could serve as one of the advantages of midventral approach particularly when dealing with nonelective laparotomy in which the patient hematocrit reading is below normal range. The packed cell volume (PCV) of the flank approach decreased significantly one week after surgery when compared with midventral approach; this could be due to high intraoperative hemorrhage recorded. This finding was in line with the finding of [20, 21], both in a study involving laparotomy with goat; they noted that remarkable hematocrit decreased after surgery with significant difference. [8] also reported significant decrease in PCV in postoperative abdominal surgery in bovine.

Higher values of total white blood cells count and lymphocytes count were recorded in midventral approach at the second week after surgery with significant difference when compared with the flank approach and this could be attributed to high persistent chronic inflammatory response in the course of tissue repair or it could be due to surgical stress because midventral approach is more stressful in relation to surgical positioning than lateral recumbency. Our finding is also in line with those of [20, 21] who also recoded elevated values leukocytes count. But [8] noticed an average total leukocytes value within normal physiologic range after abdominal surgery in dairy cows. Percentage platelets critical value recorded was higher in midventral approach; this could be due to lesser whole blood loss observed intraoperatively as decrease in whole total blood volume leads to gross interference of the different components of the blood cells including platelets. This may also serve as an advantage in midventral approach because the higher the platelets critical values, the quicker the chances of blood clotting response.

There were slight variations of means subjective healing interval of the two approaches but without significant difference (*P* = 0.643), with the flank approach having higher means number of days (13 ± 1.14) to complete surgical wound healing when compared with 12.4 ± 0.5 mean days for midventral approach. The slight variation of days of healing interval might be due to surgical site interference with the object coming contact with the surgical wound as reported by [22, 23], as the chance of surgical site contact with surrounding object is higher in flank laparotomy site compared to midventral site. The variation could also be a result of other local factors that affect wound healing like oxygenation, foreign body contact with the surgical wound, and venous insufficiency as reported by [23].

## 5. Conclusion

It was concluded that the midventral laparotomy approach can be safely and conveniently performed without fear of clinical complications in goats. When correctly performed, it will offer less intraoperative hemorrhage and postoperative tissue reactions.

We recommend the use of midventral laparotomy approach for routine abdominal surgery in goats as an alternative to flank approach. Further study on pregnant goats to see whether midventral abdominal incisional closure can withstand pressure of gravid uterus also needs to be conducted.

## Figures and Tables

**Table 1:** Criteria used to score intraoperative and postsurgical complications.

Outcome		Scores	
	0	1	2
Haemorrhage	None	Mild	Severe
Seroma	None	Mild	Severe
Wound fistula	None	Mild	Severe
Incisional hernia	None	Mild	Severe

**Table 2:** Postsurgical wound assessment score of flank and midventral approaches at 18–24 hours and 10 days (mean ± SD).

Parameters	Groups	Scores	
		18–24 hrs after surgery	10–14 days after surgery
Discharge	FA	0.80 ± 0.45	0.00 ± 0.00
	MVA	0.80 ± 0.84	0.00 ± 0.00
Swelling	FA	1.80 ± 0.45	0.50 ± 0.56
	MVA	2.00 ± 0.00	0.80 ± 0.45
Erythema	FA	1.40 ± 0.55^a^	0.25 ± 0.50^a^
	MVA	0.80 ± 0.45^b^	0.00 ± 0.00^b^
Dehiscence	FA	0.00 ± 0.00	0.25 ± 0.50^a^
	MVA	0.00 ± 0.00	0.00 ± 0.00^b^

^ab^Pair of means bearing different superscript are significantly different (*P* < 0.05).

**Table 3:** Intra- and postsurgical complications scores of flank and midventral approaches (mean ± SD).

Parameters	Groups	Scores
Intraoperative complication		
Haemorrhage	FA	1.40 ± 0.55
	MVA	1.00 ± 0.70
Postoperative complications		
Incisional hernia	FA	0.00 ± 0.00
	MVA	0.00 ± 0.00
Seroma	FA	0.00 ± 0.00
	MVA	0.00 ± 0.00
Wound fistula	FA	0.00 ± 0.00
	MVA	0.00 ± 0.00

There is no significant difference (*P* > 0.05).

**Table 4:** Total leucocytes and differential leucocytes counts before and after surgery of the flank and midventral approaches (mean ± SD).

Parameters	Groups	Mean scores			
		Before surgery	18–24 hrs after surgery	One week after surgery	Two weeks after surgery
Total WBC (×10^3^/*μℓ*)	FA	25.48 ± 4.19	37.70 ± 3.90	34.93 ± 3.12^a^	32.98 ± 5.28^a^
	MVA	33.86 ± 9.96	50.52 ± 16.32	51.08 ± 5.07^b^	45.62 ± 6.85^b^
Granulocytes (×10^3^/*μℓ*)	FA	11.10 ± 3.69	13.24 ± 3.45	10.23 ± 5.72	13.85 ± 5.33
	MVA	11.38 ± 4.41	20.90 ± 10.51	18.62 ± 5.07	15.06 ± 3.52
Lymphocytes (×10^3^/*μℓ*)	FA	11.74 ± 3.27	19.16 ± 2.61	21.33 ± 8.22^a^	15.20 ± 5.05^a^
	MVA	33.86 ± 3.40	24.06 ± 7.37	28.32 ± 11.98^b^	25.48 ± 6.00^b^
Monocytes (×10^3^/*μℓ*)	FA	2.60 ± 0.89	4.08 ± 1.21	3.35 ± 0.66	3.88 ± 0.66
	MVA	4.14 ± 1.02	5.60 ± 1.54	4.12 ± 0.44	5.06 ± 3.52

^ab^Pair of means bearing different superscript are significantly different (*P* < 0.05).

**Table 5:** Platelet characteristics before and after surgery of the two approaches (mean ± SD).

Parameters	Groups	Mean scores			
		Before surgery	18–24 hrs after surgery	One week after surgery	Two weeks after surgery
Platelets (×10^3^/*μℓ*)	FA	287.20 ± 123.58	375.60 ± 99.58	369.95 ± 144.66	269.75 ± 128.18
	MVA	351.40 ± 75.20	416.60 ± 94.88	376.20 ± 90.78	444.40 ± 149.93
Platelets critical value (%)	FA	0.16 ± 0.07	0.21 ± 0.06	0.21 ± 0.08	0.15 ± 0.04^a^
	MVA	0.20 ± 0.04	0.24 ± 0.05	0.22 ± 0.03	0.25 ± 0.08^b^
Mean platelets volume (*fℓ*)	FA	5.60 ± 0.14	5.68 ± 0.22	5.60 ± 0.09	5.55 ± 0.24
	MVA	5.72 ± 0.09	5.74 ± 0.08	5.72 ± 0.22	5.68 ± 0.13
Platelets dimension width (*fℓ*)	FA	683.90 ± 0.37	684.80 ± 0.29^a^	684.30 ± 0.05	684.30 ± 0.47
	MVA	684.26 ± 0.13	684.22 ± 0.20^b^	684.2 ± 0.18	684.12 ± 0.18

^ab^Pair of means bearing different superscript are significantly different (*P* < 0.05).

**Table 6:** Erythrocytic indices before and after surgery of the two approaches (mean ± SD).

Parameters	Groups	Mean scores			
		Before surgery	18–24 hrs after surgery	One week after surgery	Two weeks after surgery
RBC (×10^6^/*μℓ*)	FA	12.32 ± 1.35	12.79 ± 1.23	12.23 ± 1.32	12.10 ± 2.07
	MVA	13.13 ± 0.51	13.69 ± 0.52	13.36 ± 0.85	13.03 ± 1.05
PCV (%)	FA	21.92 ± 2.56	24.66 ± 5.24	16.15 ± 2.85^a^	22.75 ± 5.98
	MVA	25.22 ± 1.19	25.90 ± 1.15	25.72 ± 4.37^b^	23.84 ± 3.07
Haemoglobin (g/d)	FA	8.12 ± 1.36	8.98 ± 2.25	8.63 ± 1.51	8.68 ± 2.19
	MVA	9.16 ± 0.43	9.84 ± 0.59	9.86 ± 1.28	9.30 ± 1.36
Mean corpuscular volume (*fℓ*)	FA	17.72 ± 2.56	19.08 ± 2.37	17.58 ± 0.88	18.58 ± 1.98
	MVA	19.10 ± 2.09	18.06 ± 0.57	14.10 ± 2.09	18.20 ± 1.13
Mean corpuscular haemoglobin (pg)	FA	6.50 ± 0.42	6.88 ± 0.95	6.78 ± 0.50	7.00 ± 1.13
	MVA	6.92 ± 0.04	7.13 ± 0.26	7.37 ± 0.61	7.04 ± 0.48
Mean corpuscular haemoglobin con. (g/L)	FA	36.80 ± 2.16	36.26 ± 3.50	38.5 ± 1.94	37.36 ± 2.18
	MVA	36.32 ± 1.91	37.96 ± 1.90	38.58 ± 3.12	38.94 ± 1.82
RBC distribution width (%)	FA	30.18 ± 4.71	32.00 ± 4.37	30.98 ± 4.86	29.80 ± 6.19
	MVA	32.18 ± 1.26	34.48 ± 1.96	33.40 ± 2.23	32.92 ± 2.72

^ab^Pair of means bearing different superscript are significantly different (*P* < 0.05).
